# Early program participation and treatment retention in outpatients with gambling disorder: a real-world retrospective study in Japan

**DOI:** 10.1186/s13722-026-00689-9

**Published:** 2026-06-15

**Authors:** Hiroaki Fukui, Ryohei Takada, Yuki Noriyama, Yuki Nishi, Shintaro Araki, Fumimaro Doi, Minobu Ikehara, Ryo Mizui, Noriya Yamamoto, Takashi Okada

**Affiliations:** 1 Department of Psychiatry, Fukko-kai Tarumi Hospital, 566 Nishimori, Oshibedani-cho, Nishi-ku, Kobe City, Hyogo 651-2202 Japan; 2 Department of Psychiatry, Nara Medical University, School of Medicine, 840 Shijo-cho Kashihara, Nara, 634-8521 Japan

**Keywords:** Gambling disorder, Cognitive-behavioral therapy, Outpatient treatment, Treatment retention, Survival analysis

## Abstract

**Background:**

Real-world evidence on outpatient program participation and time to treatment discontinuation (treatment retention) in patients with gambling disorder in Japan remains limited. In this study, we characterized baseline clinical and sociodemographic profiles of outpatients receiving care for gambling disorder and described patterns of engagement, including treatment retention, in a group program.

**Methods:**

We retrospectively enrolled outpatients with gambling disorder who first visited Tarumi Hospital between April 2019 and December 2023 and followed them up until December 2024. Program participation referred to attendance at a structured cognitive behavioral therapy (CBT)-based group program designed as 24 sessions over 1 year, provided in addition to usual outpatient care. The primary analysis used a Cox proportional hazard analysis, with program participation as a time-dependent covariate; a 30-day landmark analysis was used to evaluate retention after day 30 among patients retained in the program for at least 30 days. Abstinence at the final observation was defined as a self report of no gambling in the preceding 2 weeks and was compared between the program participation and non-participation groups among patients who gambled at baseline.

**Results:**

The program was initiated for 82 patients within 30 days of their first hospital visit, and 55 patients did not participate in the program. By day 30, retention was higher in the participation group than in the non-participation group (89.0% vs. 45.5%). Program participation was associated with a lower estimated hazard of discontinuation (hazard ratio = 0.64, 95% confidence interval 0.40–1.03; *p* = 0.067), but the estimate was imprecise and compatible with no difference. Among patients retained beyond day 30, retention after day 30 did not differ between groups (log-rank *p* = 0.886). Abstinence was higher in the participation group than in the non-participation group among patients who discontinued treatment within 3 months but not among those who continued treatment for ≥ 3 or ≥ 6 months.

**Conclusions:**

Early participation in a structured CBT-based program may be associated with a longer observed retention duration, with divergence occurring within 30 days. Strengthening clinical contact soon after the first visit may be a practical priority; prospective studies with standardized severity and motivational measures are warranted.

**Supplementary Information:**

The online version contains supplementary material available at 10.1186/s13722-026-00689-9.

## Background

Gambling is defined as an activity that involves placing something of value at risk, hoping to gain something of greater value [1], and it is a common form of leisure activity across cultures. Although gambling can contribute to regional economic benefits, for some individuals, it can progress into gambling disorder (GD), which is defined in the Diagnostic and Statistical Manual of Mental Disorders, Fifth Edition (DSM-5) criteria as a persistent, recurrent pattern of gambling associated with substantial distress or impairment [2]. International epidemiological studies have used various standardized instruments and cut-off timelines to estimate problem and pathological gambling, and their 12-month prevalence has been reported to range from 0.12% to 5.8% [3]. In Japan, the prevalence of individuals suspected as having gambling disorder based on the Problem Gambling Severity Index was estimated as 1.6% [4]. Although direct international comparisons are limited by differences in instruments and cut-off timelines, this estimate is likely not exceptionally high within the internationally-reported range. Japan is characterized by its exceptionally easy access to gambling opportunities. In addition to government-operated gambling in the country, such as horse racing, motorboat racing, and bicycle racing, pachinko and pachislot (electronic gaming machines: EGMs) have become widespread in Japan, and approximately 60% of all EGMs worldwide are installed in Japan [5]. The country’s gambling market is considered the third largest in the world, following China and the United States [6]. In particular, pachinko and pachislot account for the majority of the electronic games on the market, costing up to 14.6 trillion Japanese yen (JPY) [4] and have become forms of gambling that are deeply embedded in the daily lives of Japanese. Thus, although the estimated prevalence of problem and pathological gambling in Japan does not appear to be exceptionally high, the large market size and widespread accessibility of pachinko and pachislot have created a unique gambling environment in the country. This widespread accessibility may provide basis for gambling-related effects to extend beyond individuals with severe gambling addiction to the general population. Recently, the concept of gambling-related harms (GRHs) is increasingly gaining global recognition. GRHs widely include financial, interpersonal, emotional, health, and social impacts that people who gamble, their families, close associates, and the wider community may experience [7]. Several international studies have reported that GRHs can occur among both people with problem gambling and without GD (non- or low-risk individuals with problem gambling) [8, 9]. In Japan, although people at high risk of GRH experience severe harm at the individual level, over 60% of people who experience GRHs at the population level are people without GD [10]. Thus, the diversity observed among people without GD—shaped by Japan’s high accessibility to gambling—appears to be the primary source of societal burden, highlighting a societal challenge. Accordingly, comprehensive interventions that target the broader gambling population and not only those at high risk of GRH, are needed. This is consistent with the gambling policies and public health strategies being established in many countries.

Psychosocial treatments are the mainstay of GD treatment. Recent systematic reviews and meta-analyses have supported CBT-based approaches as evidence-supported interventions for reducing gambling-related symptoms and behavior, particularly at posttreatment. However, the evidence regarding longer-term effects, active treatment components, and effectiveness in routine clinical settings remains heterogeneous [11–13]. Disengagement from GD treatment often occurs during the earliest phase of care, particularly within the first several sessions [14, 15]. Previous studies have examined a broad set of potential predictors of treatment retention or dropout, including sociodemographic factors, such as age, sex, marital status, employment, and socioeconomic status; gambling-related and clinical factors, such as gambling severity, gambling-related debt, psychiatric comorbidity, psychological distress, alcohol or substance use, and social support; and treatment-related factors, such as readiness to change and treatment exposure. However, recent reviews have suggested that few predictors have been consistently established and their reported findings are context dependent, partly because definitions of dropout, treatment settings, and available clinical variables differ across studies [16, 17]. In Japan, the clinical literature on GD has been largely based on multicenter studies conducted in large hospitals or specialized addiction clinics, typically using prospective cohort designs to describe patient characteristics and psychological correlates [18–20]. These studies have shown that GD severity among patients with GD in Japanese treatment settings is associated with cognitive distortions, impulsivity, antisocial personality disorder, and psychiatric comorbidities, including behavioral addictions [18–20]. These designs often require patient consent and structured follow-up, which may lead to the selection of patients with higher treatment engagement than that of those encountered in routine outpatient practice. In the Japanese context, where gambling is readily accessible, clinicians may also see patients with heterogeneous severity and varying readiness to continue treatment or participate in structured programs. Furthermore, studies that have evaluated outpatient program outcomes—particularly effectiveness and retention—as primary endpoints appear limited, and we are unaware of such reports from Japan.

Against this background, a retrospective analysis of clinical data from our hospital, encompassing the diversity of patient characteristics and treatment motivation, may provide insights into real-world clinical practice, thereby complementing existing evidence and improving the understanding of GD treatment in routine care. In particular, comparing patients who did and did not participate in the treatment program within the same clinical setting allows for an evaluation of the clinical significance of program participation. In this real-world retrospective study, we characterized baseline clinical and sociodemographic profiles of outpatients receiving care for GD and described patterns of engagement, including retention in care (time from the first visit to treatment discontinuation). We also summarized chart-documented gambling status at the final follow-up, including self-reported abstinence in the preceding 2 weeks among those who reported gambling at baseline.

## Methods

### Study design

This was a retrospective observational study. The study was approved by the Tarumi Hospital Ethics Committee (approval number: T24-00) in June 2024 and conducted in accordance with the Declaration of Helsinki. Study information was publicly disclosed, and an opt-out procedure was implemented in accordance with institutional policy. This study was designed and reported in accordance with the Strengthening the Reporting of Observational Studies in Epidemiology statement.

### Participants

This retrospective study included outpatients who visited Tarumi Hospital between April 1, 2019 and December 31, 2023 and met the DSM-5 for GD. Patients who had previously been diagnosed with GD at another institution and had received treatment or participated in a treatment program, as well as those with a history of participation in Gamblers Anonymous prior to the study, were excluded. The observation period extended to December 31, 2024.

To focus on early engagement in routine care, we defined three groups based on the timing of program participation: participation group (the CBT-based program was initiated within 30 days of the first visit), non-participation group (did not participate in the program during the observation period and received only usual outpatient care), and delayed participation group (program was initiated after 30 days). The main comparative analyses were restricted to the early participation and non-participation groups. Data on the delayed participation group are presented descriptively in Additional file 1 and were excluded from the main comparative analyses. Delayed initiation may reflect processes occurring during ongoing usual outpatient care after the first visit, such as changes in motivation, clinical status, gambling-related problems, life circumstances, or treatment planning rather than reflect early engagement alone. Including these patients in either the early participation or non-participation group could therefore have introduced clinical heterogeneity and would have required a separate analytical framework.

At the first outpatient visit, the attending psychiatrist encouraged all patients to participate in the treatment program; however, participation was voluntary. We included all consecutive eligible outpatients and extracted their data from electronic medical records using a predefined template. Variables required for the primary and secondary analyses were available for all included patients.

### Treatment program

Usual outpatient care for GD at our hospital included assessment of psychiatric status, provision of supportive psychotherapy, monitoring of gambling-related and psychosocial problems, brief psychoeducation about GD, and pharmacological treatment when clinically indicated for comorbid psychiatric symptoms. Patients in the participation group received usual outpatient care plus the structured CBT-based group program, whereas those in the non-participation group received usual outpatient care without participating in the structured program. The CBT-based program for GD was conducted twice a month, with each session lasting approximately 90 min. One course consisted of 24 sessions delivered over 1 year, and attendance at every session was not mandatory. Each session included approximately 15 participants and was primarily facilitated by two psychiatric social workers. The program was delivered in a group format using three structured workbook types, which were completed sequentially throughout the course. The workbook content, which was informed by established Japanese manuals for gambling problems, including STEP-G, SWITCH, and SAT-G, focused on psychoeducation, cognitive restructuring, and relapse prevention related to gambling behavior. Program participants attended their regular outpatient visits on the same days as the program sessions [21–23]. Although the program was designed with gambling abstinence as its primary therapeutic goal, clinical support was not limited to abstinence alone. Depending on each patient’s clinical situation and readiness to change, some patients also worked toward reducing gambling behavior or improving control over gambling-related problems. Therefore, abstinence was analyzed as a chart-documented clinical outcome at the final observation point rather than as the only indicator of program completion or treatment success.

### Measures

We extracted baseline characteristics from electronic medical records and categorized them as gambling-related, sociodemographic, or clinical. Gambling-related variables included the types of gambling activities, age at gambling onset, presence of gambling behavior at the first visit (defined as engagement in any gambling activity within the 2 weeks prior to the first visit), duration of gambling behavior (defined as the time from gambling onset to the first visit), amount of gambling-related debt (recorded in units of 10,000 JPY). Sociodemographic characteristics included age, sex, marital status, living with family members, employment status, education, and money management status. Clinical characteristics included the presence of alcohol use disorder, drug use disorder, other psychiatric comorbidities, and history of suicide attempts. These candidate variables were selected a priori based on clinical relevance, the prior literature on dropout and retention in gambling treatment, and availability of data in routine medical records. They were intended to cover clinically relevant domains, including sociodemographic characteristics, gambling-related burden, psychiatric comorbidity and related clinical risks, and treatment-related factors. Variables were not selected on the basis of statistical significance in the present dataset. Standardized gambling severity measures, such as the Problem Gambling Severity Index or DSM-5 symptom counts, were not systematically collected during routine clinical care. Clinical severity was assessed through routine psychiatric interviews and chart documentation.

The primary outcome was treatment retention, defined as the duration from the first visit to either treatment discontinuation or censoring. Program sessions were scheduled on the same days as outpatient psychiatrist visits to ensure that the program participants did not attend additional outpatient visits on separate days. In the participation group, patients were scheduled every 2 weeks for outpatient visits in conjunction with program sessions. Therefore, treatment discontinuation was defined as failure to attend consecutive program sessions for more than 2 months since the last attended session. In the non-participation group, follow-up outpatient visits were scheduled at intervals of up to 3 months, and treatment discontinuation was defined as missing a scheduled appointment with a period of more than 3 months since the last outpatient visit. As part of routine clinical practice, follow-up schedules differed between the program participation and non-participation groups. Therefore, treatment discontinuation was operationally defined using group-specific criteria that reflected the actual intervals of clinical contact in each group. For survival analyses, the date of the last attended session or outpatient visit was considered the event date for treatment discontinuation. Censoring was defined as the date of the last outpatient visit for patients who formally completed treatment, were transferred to another medical institution, or continued treatment until the end of the observation period. Program initiation time was defined as the number of days from the first visit to the first attended program session.

Secondary outcomes were evaluated as follows: (1) baseline characteristics were compared between the participation and non-participation groups; (2) treatment retention rates at 30 days and 3, 6, and 12 months from the first visit were compared between groups; (3) at 3 and 6 months from the first visit, patients were classified as retained or discontinued, and factors associated with treatment retention were examined; and (4) gambling abstinence rates were assessed. Abstinence was defined as no gambling within the 2 weeks prior to assessment, based on chart documentation. Gambling behavior at the first visit and final observation point (including both treatment discontinuation and censoring) was confirmed. Patients were stratified according to treatment duration (retained for ≥ 3 months, ≥ 6 months, or discontinued within 3 months). The 3-month timeline was selected because it corresponded to the operational definition of treatment discontinuation in usual outpatient care, in which follow-up visits were scheduled at intervals of up to 3 months. The 6-month timeline was used as a pragmatic time point to describe medium-term treatment retention. Within each stratum, analyses were restricted to patients who had engaged in gambling at the first visit, and abstinence rates were compared between groups. In the stratum of treatment discontinuation within 3 months, the abstinence status of patients with only one visit could not be assessed. Therefore, abstinence analyses in this stratum were restricted to patients with at least two assessments.

### Statistical analysis

Continuous variables with an approximately normal distribution are summarized as mean ± standard deviation and were compared between groups using Welch’s t-test. Non-normally distributed continuous variables are summarized as median (interquartile range [IQR]) and were compared using the Mann–Whitney U test. Dichotomous variables are expressed as n (%) and analyzed using Pearson’s chi-squared or Fisher’s exact test.

Because program initiation occurred after the first visit and the initiation time varied among patients, a Cox proportional hazards model with program participation treated as a time-dependent covariate in a counting-process (start–stop) data structure was used for the primary survival analysis. Time-varying retention patterns were visualized using Simon–Makuch curves, and between-state differences were assessed using the Mantel–Byar test.

We additionally conducted a 30-day landmark analysis restricted to patients retained for at least 30 days after the first visit, redefining time as days since day 30. Treatment retention from the landmark onward was estimated using the Kaplan–Meier method and compared between groups using the log-rank test. Landmark analysis focused on retention up to the landmark time and provides a complementary assessment of the contribution of early events. Analyses emphasizing early initiation (≤ 30 days) should be interpreted as exploratory and hypothesis-generating.

As supplementary analyses, program participation (yes/no) was also treated as a fixed exposure, and treatment retention was estimated using the Kaplan–Meier method with between-group comparisons performed using the log-rank test. Because assigning exposure as time-fixed when exposure occurs after cohort entry can introduce immortal time bias, these results are presented in the Additional Files. We also performed a sensitivity analysis in which patients with a follow-up period exceeding 1 year were administratively censored at 1 year.

Cox regression models adjusted for age at the first visit and sex were used to evaluate the association of (i) program participation as a time-dependent covariate, (ii) program participation status treated as a fixed exposure, and (iii) the number of program sessions attended by participants (reference: 1–8 sessions) with time to treatment discontinuation. Session attendance was categorized into 1–8, 9–16, and ≥ 17 sessions based on a pragmatic three-part division of the 24-session program. The 1–8 sessions group was used as the reference category because it represented the lowest level of program exposure among participants. Hazard ratios (HRs) with 95% confidence intervals (CIs) were calculated. When (quasi-)separation occurred (e.g., sparse data within a covariate level) and yielded unstable estimates, sex was omitted from the corresponding model to obtain finite estimates.

For patients classified as retained or discontinued at specific time points, baseline characteristics were compared between the groups, and multivariable logistic regression analysis was performed to test the associations between baseline variables and retention status at 3 and 6 months. Variables considered clinically relevant or that showed an association with treatment retention in the univariate analyses (*p* < 0.10) were entered into the multivariable logistic regression models. All statistical analyses were conducted using R version 4.5.1 (R Foundation for Statistical Computing, Vienna, Austria), with a two-sided significance level of *p* < 0.05.

## Results

### Baseline patient characteristics

Among 177 eligible patients, 82, 55, and 40 patients were in the participation, non-participation, and delayed participation groups, respectively. To characterize patient profiles by program participation status and to inform covariate selection for the adjusted analyses, we compared baseline characteristics between the participation and non-participation groups; these characteristics were generally similar (Table 1). The analytic sample was predominantly male (131/137, 95.6%) and consisted largely of middle-aged, employed patients living with family members. Because participation was voluntary and not randomized, these comparisons are descriptive and do not exclude residual differences due to unmeasured factors. Baseline characteristics of the delayed participation group are presented in Additional file 1.

**Table 1:** Baseline characteristics of patients in the program participation and non-participation groups

				All patients		Non-participation group		Participation group		*p*-value
				(*n* = 137)		(*n* = 55)		(*n* = 82)		
Male (n, %)				131	95.6%	51	92.7%	80	97.6%	0.219
Age (median, IQR)				35.0	30.0–41.0	35.0	30.0–41.5	35.0	30.0–40.8	0.696
Marital status (n, %)										
Married				77	56.2%	27	49.1%	50	61.0%	0.308
Divorced				13	9.5%	5	9.1%	8	9.8%	
Never married				47	34.3%	23	41.8%	24	29.3%	
Living with family (n, %)				111	81.0%	44	80.0%	67	81.7%	0.827
Employed (n, %)				117	85.4%	43	78.2%	74	90.2%	0.082
Education (n, %)										
Completed college or higher				59	43.1%	25	45.5%	34	41.5%	0.807
Completed technical school				10	7.3%	4	7.3%	6	7.3%	
Completed high school				58	42.3%	21	38.2%	37	45.1%	
Completed junior high school				10	7.3%	5	9.1%	5	6.1%	
Self-managed finances (n, %)				88	64.2%	30	54.6%	58	70.7%	0.069
Age at gambling onset (median, IQR)				20.0	18.0–25.0	19.0	18.0–23.5	20.0	18.0–26.8	0.146
Duration of gambling behavior (median, IQR)				13.0	7.0–20.0	14.0	7.5–22.5	11.5	6.2–19.0	0.196
Gambling-related debt (median, IQR), 10,000 JPY				300.0	110.0–600.0	200.0	65.0–600.0	300.0	152.5–600.0	0.115
Gambling activity (multiple answers) (n, %)										
Pachinko				91	66.4%	39	70.9%	52	63.4%	0.468
Pachislot				38	27.7%	18	32.7%	20	24.4%	0.382
Horse racing				68	49.6%	29	52.7%	39	47.6%	0.676
Boat racing				42	30.7%	16	29.1%	26	31.7%	0.891
Bicycle racing				21	15.3%	8	14.5%	13	15.9%	1.000
Foreign / stock exchange				20	14.6%	11	20.0%	9	11.0%	0.222
Casino				12	8.8%	6	10.9%	6	7.3%	0.543
Mahjong				2	1.5%	0	0.0%	2	2.4%	0.516
Lottery				2	1.5%	2	3.6%	0	0.0%	0.159
Gambling behavior at the first visit (n, %)				91	66.4%	39	70.9%	52	63.4%	0.468
Alcohol use disorder (n, %)				5	3.6%	3	5.5%	2	2.4%	0.390
Drug use disorder (n, %)				2	1.5%	2	3.6%	0	0.0%	0.159
Other psychiatric comorbidities (n, %)				22	16.0%	8	14.5%	14	17.1%	0.874
Suicide attempts (n, %)				7	5.1%	2	3.6%	5	6.1%	0.701

Continuous variables are presented as median (interquartile range, IQR) and were compared using the Mann–Whitney U test. Categorical variables are presented as n (%) and were compared using Pearson’s chi-squared or Fisher’s exact test, as appropriate. IQR, interquartile range; JPY, Japanese yen

### Treatment retention and survival analysis

Treatment retention was compared between the participation and non-participation groups. As a descriptive assessment, retention rates at prespecified time points (30 days and 3, 6, and 12 months from the first visit) were calculated and compared. Retention was consistently higher in the participation group than in the non-participation group, with the largest difference observed at 30 days (89.0% vs. 45.5%; Additional file 2).

**Fig. 1:**
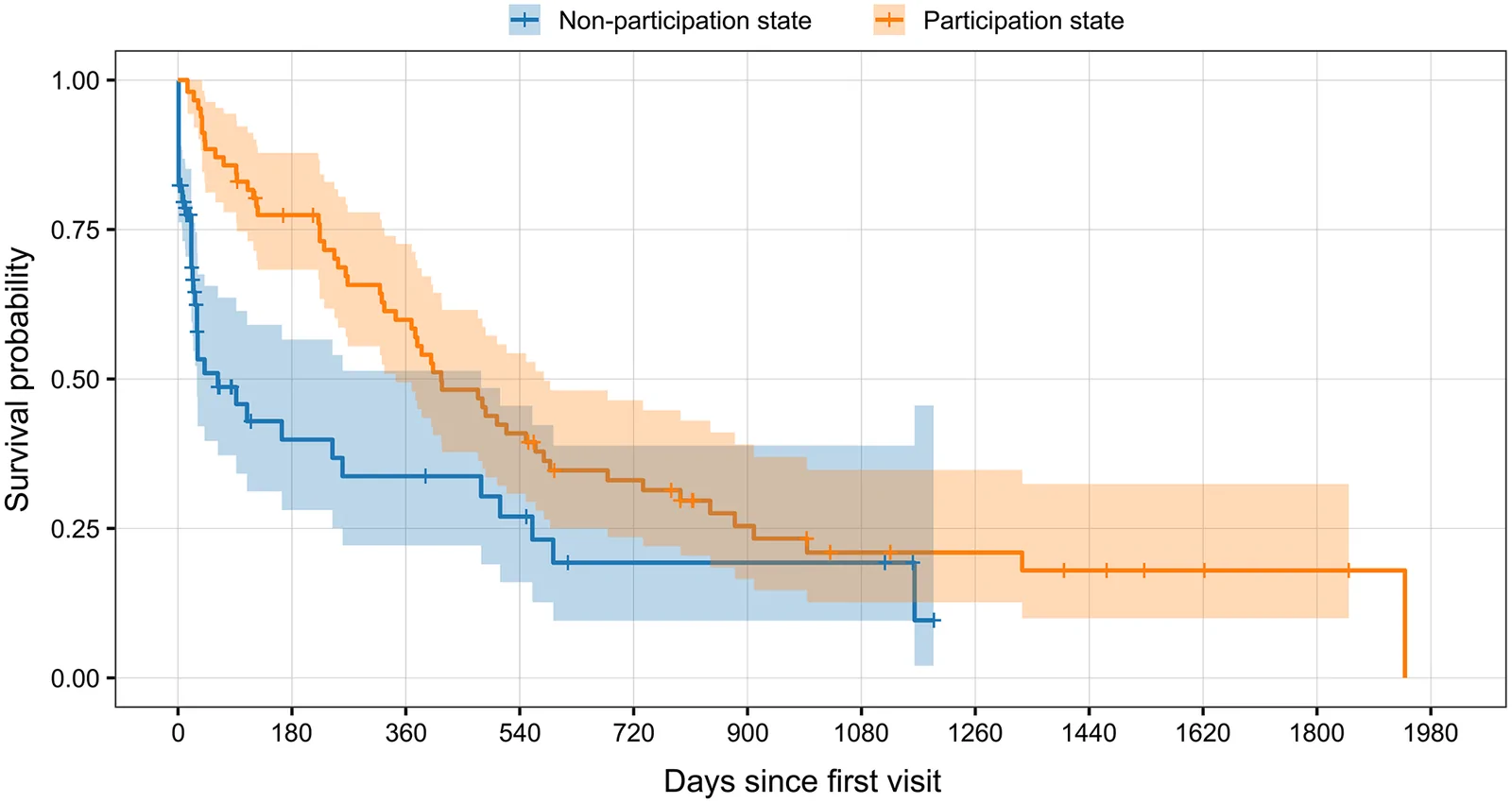
Simon–Makuch curves for treatment retention according to program participation status as a time-dependent covariate. The orange line represents the participation group, and the blue line represents the non-participation group. Shaded areas indicate 95% confidence intervals. Short vertical ticks indicate censoring. Between-group differences were evaluated using the Mantel–Byar test (*p* = 0.062)

**Fig. 2:**
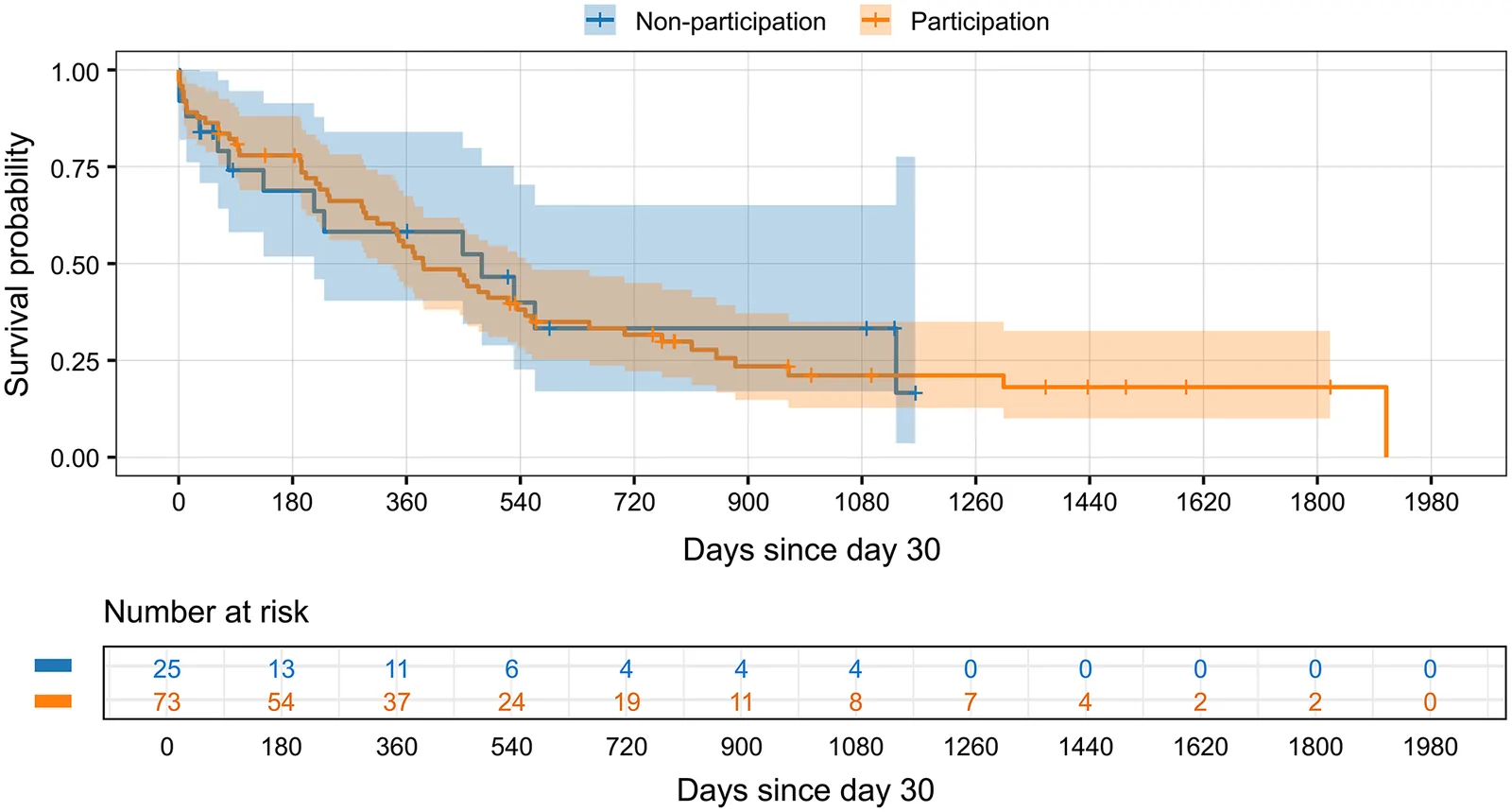
Kaplan–Meier curves for treatment retention from the 30-day landmark. The orange line represents the program participation group, and the blue line represents the non-participation group. Shaded areas indicate 95% confidence intervals. Short vertical ticks indicate censoring. The numbers of patients at risk are shown below the x-axis. Treatment retention did not differ significantly between groups (log-rank test, p=0.886)

In the primary analysis, a Cox proportional hazards model with program participation treated as a time-dependent covariate was fitted with adjustments for age and sex. The estimated HR for discontinuation associated with participation was 0.64 (95% CI 0.40–1.03; *p* = 0.067). The Simon–Makuch plot was directionally consistent with earlier separation of the curves (Fig. 1), and the Mantel–Byar test was not statistically definitive (*p* = 0.062). To examine whether the between-group difference was primarily established early after the first visit, we conducted a 30-day landmark analysis restricted to patients who remained in care for at least 30 days. From the landmark onward, Kaplan–Meier curves did not differ between groups (log-rank test, *p* = 0.886; Fig. 2), and an age-adjusted Cox model similarly showed no significant association between program participation and the hazard of discontinuation (age-adjusted HR = 1.08, 95% CI 0.60–1.94, *p* = 0.81); sex could not be estimated due to (quasi-)separation and was therefore excluded. Kaplan–Meier curves and Cox models with program participation as a fixed exposure, as well as the corresponding analyses with follow-up censored at 1 year, are presented in Additional files 3 and 4. Among program participants, retention by the number of sessions attended (1–8, 9–16, and ≥ 17) is shown in Additional file 5.

### Factors associated with treatment retention at specific time points

At each predefined time point, patients were classified as retained or discontinued, and factors associated with treatment retention were examined (Additional files 6 and 7). Program participation was independently associated with higher retention at 3 months (adjusted OR = 8.39, 95% CI 3.65–19.33; Table 2) and 6 months (adjusted OR = 6.80, 95% CI 2.99–15.47; Table 3). Other independent correlates included other psychiatric comorbidities at 3 months (*p* = 0.020) and suicide-attempt history (*p* = 0.038) and gambling at the first visit (inverse) at 6 months (*p* = 0.030).

**Table 2:** Multivariable logistic regression analysis of factors associated with treatment retention at 3 months

Variable			Adjusted OR	95% CI	*p*-value
Participated in the treatment program			8.39	3.65–19.33	< 0.001
Other psychiatric comorbidities			4.95	1.28–19.13	0.020
Age (years)			0.99	0.96–1.03	0.792
Sex (male)			1.40	0.19–10.49	0.742
Debt (per 100,000 JPY increase)			1.005	0.999–1.011	0.078
Casino gambling			0.23	0.05–1.10	0.065

CI, confidence interval; OR, odds ratio; JPY, Japanese Yen. Adjusted ORs with 95% CIs are presented. Covariates included program participation, other psychiatric comorbidities, age, sex, casino gambling, and gambling-related debt, with ORs for debt expressed per 100,000 JPY increase

**Table 3:** Multivariable logistic regression analysis of factors associated with treatment retention at 6 months

Variable			Adjusted OR	95% CI	*p*-value
Participation in the treatment program			6.80	2.99–15.47	< 0.001
Age (years)			1.02	0.98–1.06	0.270
Debt (per 100,000 JPY increase)			1.005	0.999–1.010	0.102
Suicide attempts			12.81	1.15–142.98	0.038
Gambling behavior at the first visit (n, %)			0.40	0.17–0.92	0.030

CI, confidence interval; OR, odds ratio; JPY, Japanese Yen. Adjusted ORs with 95% CIs are presented. Covariates included program participation, age, history of suicide attempts, and presence of gambling behavior at the first visit, and gambling-related debt, with ORs for debt expressed per 100,000 JPY increase. Sex was excluded due to complete data separation

### Gambling abstinence rates according to treatment duration

Gambling abstinence at the final observation point, stratified by treatment duration, was compared between the participation and non-participation groups among patients who reported gambling behavior at the first visit (Table 4). In the < 3-month discontinuation stratum, abstinence was assessed only among patients with ≥ 2 clinical assessments. In the non-participation group, 18 patients who had engaged in gambling at the first visit attended only the first visit and subsequently discontinued outpatient care; therefore, they could not be evaluated for abstinence at the final observation point. Among patients evaluable for abstinence in this stratum, the abstinence proportion was higher in the participation group than in the non-participation group (12/13 [92.3%] vs. 3/9 [33.3%], *p* = 0.007). In contrast, among patients retained for ≥ 3 months (16/39 [41.0%] vs. 2/12 [16.7%], *p* = 0.174) and ≥ 6 months (11/31 [35.5%] vs. 0/8 [0%], *p* = 0.078), abstinence rates were similar between the groups. Detailed distributions of gambling behavior at the final observation point by treatment-duration strata are provided in Additional file 8.

**Table 4:** Gambling abstinence at the final observation point according to treatment duration and program participation

Treatment duration		Group		*n* (%)	*p*-value
≥ 3 months		Non-participation (*n* = 12)		2 (16.7%)	0.174
		Participation (*n* = 39)		16 (41.0%)	
≥ 6 months		Non-participation (*n* = 8)		0 (0.0%)	0.078
		Participation (*n* = 31)		11 (35.5%)	
< 3 months		Non-participation (*n* = 9)		3 (33.3%)	0.007
		Participation (*n* = 13)		12 (92.3%)	

Abstinence rates among patients who had engaged in gambling at the first visit, stratified by treatment duration, were compared between the program participation and non-participation groups. In the stratum of treatment discontinuation within 3 months, analyses were restricted to patients with at least two assessments because abstinence status could not be assessed in single-visit cases. Data are shown as n (%). Fisher’s exact test was used for group comparisons

## Discussion

This retrospective study of outpatients with gambling disorder suggests that engagement in a structured CBT-based outpatient program may be associated with longer observed treatment retention than that observed with usual outpatient care alone, while underscoring the importance of early-period processes. Descriptive retention rates were consistently higher in the participation group than in the non-participation group at 30 days and 3, 6, and 12 months, with a particularly large difference already evident at 30 days (89.0% vs. 45.5%). In the primary analysis, with program participation treated as a time-dependent covariate, participation was associated with a lower estimated hazard of discontinuation, but the estimate was imprecise and compatible with no difference (adjusted HR = 0.64, 95% CI 0.40–1.03; *p* = 0.067), and the Simon–Makuch plot and Mantel–Byar test indicated a directionally consistent, although not statistically definitive, separation. Importantly, in the 30-day landmark analysis restricted to patients who remained in care for at least 30 days after the first visit, the between-group difference was substantially attenuated and was no longer clearly evident, suggesting that the divergence between groups may have largely been established during the early post–first-visit period, particularly within the first month. In addition, because routine follow-up intervals differed between groups, early separation may reflect differences in the frequency and continuity of clinical contact under routine scheduling, in addition to program participation. Taken together, these findings indicate that the early post–first-visit period is a high-risk window for treatment discontinuation and that maintaining clinical contact soon after the initial visit may be a practical implementation priority in routine care. Therefore, when added to usual outpatient care, the CBT-based program may have provided a structured opportunity to maintain frequent and regular clinical contact during the early phase of outpatient treatment. Previous studies have reported that treatment attendance for GD declines rapidly over the first several sessions, with a non-negligible proportion of very early dropouts [14, 15]. Similarly, real-world outpatient service data from New Zealand (2014–2023; *n* = 18,642) indicate substantial early attrition: 27.7% of clients attended only one session, and 36.8% attended six or more sessions [16]. Campos et al. also reported that dropout was highest after intake and decreased across subsequent sessions among people who gamble and exhibit depressive symptoms [15]. The present findings therefore align with this well-recognized challenge of “early dropout clustering,” while extending it to a Japanese routine outpatient setting in which early participation in a structured CBT-based group program was added to usual outpatient care.

Gambling abstinence at the final observation differed by treatment duration. Among patients who discontinued treatment within 3 months and were evaluable for abstinence, the participation group compared with the non-participation group showed a higher proportion of abstinence, whereas no statistically significant between-group differences were observed among those retained for at least 3 or 6 months. These findings require cautious interpretation. Abstinence was defined based on self reports of “no gambling in the 2 weeks immediately preceding the last observation” on a chart documentation, which may not adequately capture longer-term dynamics, such as sustained remission and relapse. Moreover, because the participation and non-participation groups differed in expected visit frequency, the timing and context of the last observation (i.e., the last point at which gambling status could be confirmed) may not be comparable between the groups, potentially reducing the comparability of abstinence rates defined using a 2-week window. This concern is particularly relevant to the < 3-month discontinuation stratum, in which 18 patients in the non-participation group had assessments for only the first visit; therefore, they could not be evaluated for abstinence at the final observation point. Because all non-evaluable patients in this stratum were in the non-participation group, the apparent between-group difference in abstinence among patients who discontinued treatment within 3 months should be interpreted with particular caution. In addition, gambling behavior after treatment discontinuation was not examined in this cohort; therefore, abstinence at the final observation may not reflect longer-term outcomes. Accordingly, the stratified abstinence results should be regarded as descriptive and hypothesis-generating rather than evidence of a causal effect.

The early-phase pattern observed in this study remains clinically relevant because structured interventions delivered over a short period may coincide with changes in behavioral outcomes. Previous studies have reported associations between brief interventions for problem gambling and improvements in gambling behavior or symptom severity [24, 25]. A meta-analysis of CBT, motivational interviewing, and personalized feedback suggested that treatment dose relates to effect size at the end of treatment, while some studies have reported comparable outcomes between single-session and multi-session formats; thus, the optimal “necessary” treatment dose remains uncertain [26]. These observations support the need to design and evaluate brief or early-phase focused interventions.

Multivariable analyses further suggested that factors associated with retention may differ across time durations. Psychiatric comorbidity showed a stronger association with retention at 3 months, whereas a history of suicide attempt showed a stronger association with retention at 6 months. Previous studies have suggested that treatment retention may relate to demographic and socioeconomic factors as well as psychological characteristics, such as depression, psychological distress, and alcohol use. However, the findings have not been fully consistent and may vary depending on whether severity, debt, and other clinical factors are included as independent variables [17, 27]. The associations with comorbidity and suicide-related risk in the present study are consistent with an interpretation that higher-risk patients may receive more intensive monitoring and support in routine care, which could contribute to sustained engagement. Nonetheless, estimates for suicide-attempt history had wide CIs, indicating substantial uncertainty, and the possibility of sparse-data bias and residual confounding should be considered. Prospective studies using standardized measures of psychiatric symptoms, suicidality, and gambling severity are needed.

Several limitations in this study warrant consideration. First, this single-center retrospective design limits generalizability and precludes causal inference. In addition, because we excluded patients for whom the program was initiated after 30 days, our findings primarily inform early program participation and may not be generalizable to later initiators. Although the male predominance in this study is consistent with Japanese epidemiological findings, the marked sex imbalance in this cohort limits generalizability. Furthermore, treatment engagement and retention among women with GD in Japan may differ from those observed in this study. Second, because participation was voluntary, unmeasured factors, such as baseline motivation or readiness to change, standardized gambling-severity measures, and family or social support, may have influenced both program uptake and subsequent retention, leaving residual confounding. Third, because the expected follow-up intervals differed between groups, treatment discontinuation was operationally defined using group-specific criteria; this difference may influence both the timing of discontinuation identification and determination of the final observation point, thereby affecting comparability of the retention endpoint between the groups. The shorter discontinuation window in the program group may lead to earlier event ascertainment, potentially biasing the estimated retention advantage of the program toward the null. Moreover, because the program participants had approximately fortnightly contacts whereas those receiving usual outpatient care alone had contact intervals of up to 3 months, visit frequency itself may have acted as a structural retention mechanism. Thus, the observed association may partly reflect the intensity and continuity of clinical contact rather than the specific content of the CBT program alone. Fourth, the modest sample size relative to the number of covariates may have limited the stability of the multivariable models. Some baseline clinical variables, such as alcohol and drug use disorders, had very small cell sizes and were therefore difficult to evaluate as candidate predictors. Among variables included in the final models, sparse data for history of suicide attempts contributed to imprecision, as reflected by the wide CI. In addition, separation-related difficulty in estimating sex effects was observed in certain models. Because several baseline variables and outcomes were examined exploratively without adjustment for multiple testing, some of the statistically significant findings may reflect findings due to chance. Therefore, some multivariable estimates and exploratory between-group comparisons should be interpreted cautiously. Finally, self-discontinuation can reflect heterogeneous pathways, including perceived improvement, burden of visits and external constraints, reduced motivation, relapse, or avoidance. This is consistent with a previous study suggesting that some patients who discontinue psychotherapy after the first few sessions may represent early treatment responders rather than treatment failures [15]. While cases of mutually-agreed termination were identifiable, the reasons underlying self-discontinuation could not be consistently determined. Therefore, treatment retention should not be interpreted directly as superiority of outcome or strength of treatment effect but rather as an indicator of the continuity of clinical contact. In routine outpatient services, systematically recording the reasons for treatment discontinuation may help distinguish improvement-related termination or early treatment response from disengagement, relapse, avoidance, or external barriers to attendance.

## Conclusions

In real-world outpatient data from patients with GD, program participation may be associated with longer observed treatment retention, with most between-group divergence occurring within the first 30 days after the initial visit. In the time-dependent Cox analyses, participation was associated with a lower estimated hazard of treatment discontinuation (HR = 0.64, 95% CI 0.40–1.03; *p* = 0.067), but the estimate was imprecise and compatible with no difference. Consistent with this pattern, 30-day landmark analyses showed no retention difference between groups after day 30 among patients who remained in care beyond 30 days. These findings support the practical importance of early structured clinical contact. Future prospective studies should incorporate standardized assessments of baseline motivation and severity and extend follow-up beyond treatment discontinuation to evaluate whether brief interventions and stepped-care approaches improve retention and longer-term outcomes, including sustained abstinence and relapse prevention. 

## Supplementary Information

Below is the link to the electronic supplementary material.

**Supplementary Material 1: Additional file 1:** Baseline characteristics of patients in the delayed participation group. Additional file 1 presenting baseline demographic, clinical, and gambling-related characteristics of the delayed participation group.

**Supplementary Material 2: Additional file 2:** Treatment retention rates at 30 days and 3, 6, and 12 months by program participation status. Additional file 2 summarizing treatment retention rates at 30 days and 3, 6, and 12 months by program participation status, with between-group comparisons.

**Supplementary Material 3: Additional file 3:** Kaplan–Meier curves for treatment retention by program participation. Kaplan–Meier curves of treatment retention stratified by program participation status, showing 95% confidence intervals, censoring marks, and numbers at risk, with the log-rank test result and age- and sex-adjusted Cox model estimates.

**Supplementary Material 4: Additional file 4: **Kaplan–Meier curves for treatment retention censored at 1 year. Kaplan–Meier curves of treatment retention with follow-up administratively censored at 1 year, including 95% confidence intervals, censoring marks, numbers at risk, and between-group comparisons (log-rank test and age- and sex-adjusted Cox model estimates).

**Supplementary Material 5: Additional file 5:** Kaplan–Meier curves for treatment retention according to the number of program sessions attended. Kaplan–Meier curves of treatment retention among program participants stratified by number of sessions attended (1–8, 9–16, and ≥17), with numbers at risk and age-adjusted Cox model estimates.

**Supplementary Material 6: Additional file 6:** Univariate analysis of factors associated with treatment retention at 3 months. Baseline factors associated with retention at 3 months (retained vs discontinued), with p-values from Mann–Whitney U and chi-squared/Fisher’s exact tests.

**Supplementary Material 7: Additional file 7:** Univariate analysis of factors associated with treatment retention at 6 months. Baseline factors associated with retention at 6 months (retained vs discontinued), with p-values from Mann–Whitney U and chi-squared/Fisher’s exact tests.

**Supplementary Material 8: Additional file 8:** Gambling behavior at the final observation point by treatment duration and program participation. Descriptive summary of gambling behavior at the final observation point, stratified by treatment-duration category (<3 months, ≥3 months, and ≥6 months) and program participation status, reported as n (%).

## Data Availability

The data that support the findings of this study are not publicly available due to privacy and ethical restrictions related to patient data. De-identified data may be made available from the corresponding author upon reasonable request and with approval from the relevant ethics committee/institution, where applicable.

## Abbreviations

CBT: Cognitive behavioral therapy
CI: Confidence interval
DSM-5: Diagnostic and statistical manual of mental disorders, fifth edition
EGM: Electronic gaming machine
GD: Gambling disorder
GRH: Gambling-related harm
HR: Hazard ratio
IQR: Interquartile range
