# Supplementary material for: Early program participation and treatment retention in outpatients with gambling disorder: a real-world retrospective study in Japan
Source: Addict Sci Clin Pract. 2026 Jun 15;21:63. doi: 10.1186/s13722-026-00689-9 (PMC13501588; doi:10.1186/s13722-026-00689-9)
Supplement: Supplementary file 1 [file 13722_2026_689_MOESM1_ESM.docx]

Additional file 1. Baseline characteristics of patients in the delayed participation group.

|  |  |  |  | Total (n=40) | |
| --- | --- | --- | --- | --- | --- |
|  |  |  |  |  |  |
| Male (n, %) |  |  |  | 38 | 95.0% |
| Age (median, IQR) | |  |  | 34.5 | 28.0–42.5 |
| Marital status (n, %) | |  |  |  |  |
| Married |  |  |  | 19 | 47.5% |
| Divorced |  |  |  | 5 | 12.5% |
| Never married | |  |  | 16 | 40.0% |
| Living with family (n, %) | |  |  | 32 | 80.0% |
| Employed (n, %) | |  |  | 36 | 90.0% |
| Education (n, %) | |  |  |  |  |
| Completed college or higher | |  |  | 22 | 55.0% |
| Completed technical school | | |  | 1 | 2.50% |
| Completed high school | |  |  | 16 | 40.0% |
| Completed junior high school | | |  | 1 | 2.50% |
| Self-managed finances (n, %) | | |  | 29 | 72.5% |
| Age at gambling onset (median, IQR) | | |  | 21.5 | 19.5–27.0 |
| Duration of gambling behavior (median, IQR) | | | | 9.0 | 5.0–15.0 |
| Gambling-related debt (median, IQR), 10,000 JPY | | | | 400.0 | 185.0–925.0 |
| Gambling activity (multiple answers) (n, %) | | | |  |  |
| Pachinko |  |  |  | 25 | 62.5% |
| Pachislot |  |  |  | 10 | 25.0% |
| Horse racing | |  |  | 14 | 35.0% |
| Boat racing | |  |  | 11 | 27.5% |
| Bicycle racing | |  |  | 2 | 5.0% |
| Foreign / stock exchange | | |  | 1 | 2.5% |
| Casino |  |  |  | 4 | 10.0% |
| Mahjong |  |  |  | 0 | 0.0% |
| Lottery |  |  |  | 2 | 5.0% |
| Gambling behavior at the first visit (n, %) | | | | 19 | 47.5% |
| Alcohol use disorder (n, %) | | |  | 2 | 5.0% |
| Drug use disorder (n, %) | |  |  | 0 | 0.0% |
| Other psychiatric comorbidities (n, %) | | |  | 5 | 12.5% |
| Suicide attempts (n, %) | |  |  | 0 | 0.0% |

Continuous variables are presented as median (interquartile range, IQR), and categorical variables are presented as n (%).
