# Supplementary material for: Early program participation and treatment retention in outpatients with gambling disorder: a real-world retrospective study in Japan
Source: Addict Sci Clin Pract. 2026 Jun 15;21:63. doi: 10.1186/s13722-026-00689-9 (PMC13501588; doi:10.1186/s13722-026-00689-9)
Supplement: Supplementary file 2 [file 13722_2026_689_MOESM2_ESM.docx]

Additional file 2. Treatment retention rates at 30 days and 3, 6, and 12 months by program participation status.

| Time point | Non-participation group (n=55) | Participation group (n=82) | p-value |
| --- | --- | --- | --- |
| 30 days | 25 (45.5%) | 73 (89.0%) | ＜0.001 |
| 3 months | 17 (30.9%) | 63 (76.8%) | ＜0.001 |
| 6 months | 13 (23.6%) | 54 (65.9%) | ＜0.001 |
| 12 months | 11 (20.0%) | 41 (50.0%) | ＜0.001 |

Data are presented as n (%). Group comparisons were performed using the chi-squared test.
