# Supplementary material for: Early program participation and treatment retention in outpatients with gambling disorder: a real-world retrospective study in Japan
Source: Addict Sci Clin Pract. 2026 Jun 15;21:63. doi: 10.1186/s13722-026-00689-9 (PMC13501588; doi:10.1186/s13722-026-00689-9)
Supplement: Supplementary file 3 [file 13722_2026_689_MOESM3_ESM.docx]

Additional file 3. Kaplan–Meier curves for treatment retention by program participation. The orange line represents the program participation group, and the blue line represents the non-participation group. Shaded areas indicate 95% confidence intervals (CI). Short vertical ticks indicate censoring. The numbers of patients at risk are shown below the x-axis. Treatment retention was significantly longer in the participation group than in the non-participation group (log-rank test, p < 0.001). In a Cox proportional hazards model adjusted for age and sex, program participation was associated with a lower hazard of treatment discontinuation (adjusted hazard ratio = 0.46, 95% CI 0.31–0.68, p < 0.001).
