# Supplementary material for: Early program participation and treatment retention in outpatients with gambling disorder: a real-world retrospective study in Japan
Source: Addict Sci Clin Pract. 2026 Jun 15;21:63. doi: 10.1186/s13722-026-00689-9 (PMC13501588; doi:10.1186/s13722-026-00689-9)
Supplement: Supplementary file 5 [file 13722_2026_689_MOESM5_ESM.docx]

Additional file 5. Kaplan–Meier curves for treatment retention according to the number of program sessions attended. Kaplan–Meier curves show treatment retention among patients who participated in the treatment program, stratified by the number of program sessions attended. The blue line represents patients who attended 1–8 sessions, the orange line represents those who attended 9–16 sessions, and the green line represents those who attended ≥17 sessions. Shaded areas indicate 95% confidence intervals (CI). Short vertical ticks indicate censoring. The numbers of patients at risk are shown below the x-axis. In a Cox proportional hazards model adjusted for age, attendance at ≥17 sessions was independently associated with a significantly lower hazard of treatment discontinuation compared with that observed for attendance at 1–8 sessions (age-adjusted hazard ratio [HR] = 0.16, 95% CI 0.06–0.42, p < 0.001). In contrast, attendance at 9–16 sessions compared with attendance at 1–8 sessions was not significantly associated with treatment retention (age-adjusted HR = 0.79, 95% CI 0.39–1.60, p=0.512).
