# Supplementary material for: Early program participation and treatment retention in outpatients with gambling disorder: a real-world retrospective study in Japan
Source: Addict Sci Clin Pract. 2026 Jun 15;21:63. doi: 10.1186/s13722-026-00689-9 (PMC13501588; doi:10.1186/s13722-026-00689-9)
Supplement: Supplementary file 6 [file 13722_2026_689_MOESM6_ESM.docx]

Additional file 6. Univariate analysis of factors associated with treatment retention at 3 months.

|  |  |  |  | Discontinued | | Retained | |  |
| --- | --- | --- | --- | --- | --- | --- | --- | --- |
|  |  |  |  | by 3 months (n=57) | | at 3 months (n=80) | | p-value |
| Male (n, %) |  |  |  | 54 | 94.7% | 77 | 96.2% | 0.693 |
| Age (median, IQR) | |  |  | 34.0 | 30.0–41.0 | 36.0 | 30.0–41.0 | 0.755 |
| Marital status (n, %) | |  |  |  |  |  |  |  |
| Married |  |  |  | 31 | 54.4% | 46 | 57.5% | 0.911 |
| Divorced |  |  |  | 6 | 10.5% | 7 | 8.8% |  |
| Never married | |  |  | 20 | 35.1% | 27 | 33.8% |  |
| Living with family (n, %) | |  |  | 47 | 82.5% | 64 | 80.0% | 0.888 |
| Employed (n, %) | |  |  | 46 | 80.7% | 71 | 88.8% | 0.285 |
| Education (n, %) | |  |  |  |  |  |  |  |
| Completed college or higher | |  |  | 27 | 47.4% | 32 | 40.0% | 0.110 |
| Completed technical school | | |  | 6 | 10.5% | 4 | 5.0% |  |
| Completed high school | |  |  | 18 | 31.6% | 40 | 50.0% |  |
| Completed junior high school | | |  | 6 | 10.5% | 4 | 5.0% |  |
| Self-managed finances (n, %) | | |  | 38 | 66.7% | 50 | 62.5% | 0.748 |
| Age at gambling onset (median, IQR) | | |  | 20.0 | 18.0–23.0 | 20.0 | 18.0–25.3 | 0.678 |
| Duration of gambling behavior (median, IQR) | | | | 11.0 | 6.0–21.0 | 13.0 | 7.0–19.3 | 0.976 |
| Gambling-related debt (median, IQR), 10,000 JPY | | | | 200.0 | 70.0–450.0 | 380.0 | 190.0–725.0 | 0.006 |
| Gambling activity (multiple answers) (n, %) | | | |  |  |  |  |  |
| Pachinko |  |  |  | 42 | 73.7% | 49 | 61.3% | 0.182 |
| Pachislot |  |  |  | 17 | 29.8% | 21 | 26.2% | 0.789 |
| Horse racing | |  |  | 33 | 57.9% | 35 | 43.8% | 0.145 |
| Boat racing | |  |  | 21 | 36.8% | 21 | 26.2% | 0.255 |
| Bicycle racing | |  |  | 8 | 14.0% | 13 | 16.2% | 0.909 |
| Foreign / stock exchange | | |  | 6 | 10.5% | 14 | 17.5% | 0.371 |
| Casino |  |  |  | 8 | 14.0% | 4 | 5.0% | 0.075 |
| Mahjong |  |  |  | 1 | 1.8% | 1 | 1.2% | 1.000 |
| Lottery |  |  |  | 1 | 1.8% | 1 | 1.2% | 1.000 |
| Gambling behavior at the first visit (n, %) | | | | 40 | 70.2% | 51 | 63.7% | 0.548 |
| Participated in the treatment program　(n, %) | | | | 19 | 33.3% | 63 | 78.8% | < 0.001 |
| Alcohol use disorder (n, %) | | |  | 2 | 3.5% | 3 | 3.8% | 1.000 |
| Drug use disorder (n, %) | |  |  | 1 | 1.8% | 1 | 1.2% | 1.000 |
| Other psychiatric comorbidities (n, %) | | |  | 4 | 7.0% | 18 | 22.5% | 0.028 |
| Suicide attempts (n, %) | |  |  | 1 | 1.8% | 6 | 7.5% | 0.239 |

Patients were classified as retained at 3 months or discontinued by 3 months. Continuous variables are presented as median (interquartile range, IQR) and were compared using the Mann–Whitney U test. Categorical variables are presented as n (%) and were compared using Pearson’s chi-squared or Fisher’s exact test, as appropriate.
