# Supplementary material for: Early program participation and treatment retention in outpatients with gambling disorder: a real-world retrospective study in Japan
Source: Addict Sci Clin Pract. 2026 Jun 15;21:63. doi: 10.1186/s13722-026-00689-9 (PMC13501588; doi:10.1186/s13722-026-00689-9)
Supplement: Supplementary file 7 [file 13722_2026_689_MOESM7_ESM.docx]

Additional file 7. Univariate analysis of factors associated with treatment retention at 6 months.

|  |  |  |  | Discontinued | | Retained | |  |
| --- | --- | --- | --- | --- | --- | --- | --- | --- |
|  |  |  |  | by 6 months (n=70) | | at 6 months (n=67) | | p-value |
| Male (n, %) |  |  |  | 64 | 91.40% | 67 | 100.0% | 0.028 |
| Age (median, IQR) | |  |  | 35.0 | 29.3–41.8 | 36.0 | 30.0–40.5 | 0.760 |
| Marital status (n, %) | |  |  |  |  |  |  |  |
| Married |  |  |  | 34 | 48.60% | 43 | 57.5% | 0.162 |
| Divorced |  |  |  | 7 | 10.00% | 6 | 9.0% |  |
| Never married | |  |  | 29 | 41.40% | 18 | 26.9% |  |
| Living with family (n, %) | |  |  | 55 | 78.60% | 56 | 83.6% | 0.596 |
| Employed (n, %) | |  |  | 56 | 80.00% | 61 | 91.0% | 0.112 |
| Education (n, %) | |  |  |  |  |  |  |  |
| Completed college or higher | |  |  | 29 | 41.40% | 30 | 44.8% | 0.330 |
| Completed technical school | | |  | 7 | 10.00% | 3 | 4.5% |  |
| Completed high school | |  |  | 27 | 38.60% | 31 | 46.3% |  |
| Completed junior high school | | |  | 7 | 10.00% | 3 | 4.5% |  |
| Self-managed finances (n, %) | | |  | 45 | 64.30% | 43 | 64.2% | 1.000 |
| Age at gambling onset (median, IQR) | | |  | 20.0 | 18.0–24.8 | 20.0 | 18.0–25.5 | 0.96 |
| Duration of gambling behavior (median, IQR) | | | | 11.0 | 6.0–20.8 | 13.0 | 7.0–19.5 | 0.625 |
| Amount of gambling-related debt (median, IQR) | | | | 200.0 | 76.3–495.0 | 360.0 | 180.0–825.0 | 0.008 |
| Gambling activity (multiple answers) (n, %) | | | |  |  |  |  |  |
| Pachinko |  |  |  | 50 | 71.40% | 41 | 61.2% | 0.277 |
| Pachislot |  |  |  | 22 | 31.40% | 16 | 23.9% | 0.426 |
| Horse racing | |  |  | 36 | 51.40% | 32 | 47.8% | 0.796 |
| Boat racing | |  |  | 23 | 32.90% | 19 | 28.4% | 0.700 |
| Bicycle racing | |  |  | 9 | 12.90% | 12 | 17.9% | 0.56 |
| Foreign / stock exchange | | |  | 10 | 14.30% | 10 | 14.9% | 1.000 |
| Casino |  |  |  | 9 | 12.90% | 3 | 4.5% | 0.152 |
| Mahjong |  |  |  | 1 | 1.40% | 1 | 1.5% | 1.000 |
| Lottery |  |  |  | 2 | 2.90% | 0 | 0.0% | 0.497 |
| Gambling behavior at the first visit (n, %) | | | | 52 | 74.30% | 39 | 58.2% | 0.070 |
| Participated in the treatment program　(n, %) | | | | 28 | 40.00% | 54 | 80.6% | < 0.001 |
| Alcohol use disorder (n, %) | | |  | 2 | 2.90% | 3 | 4.5% | 0.676 |
| Drug use disorder (n, %) | |  |  | 1 | 1.40% | 1 | 1.5% | 1.000 |
| Other psychiatric comorbidities (n, %) | | |  | 8 | 11.40% | 14 | 20.9% | 0.202 |
| Suicide attempts (n, %) | |  |  | 1 | 1.40% | 6 | 9.0% | 0.059 |

Patients were classified as retained at 6 months or discontinued by 6 months. Continuous variables are presented as median (interquartile range, IQR) and were compared using the Mann–Whitney U test. Categorical variables are presented as n (%) and were compared using Pearson’s chi-squared or Fisher’s exact test, as appropriate.
