# Supplementary material for: Early program participation and treatment retention in outpatients with gambling disorder: a real-world retrospective study in Japan
Source: Addict Sci Clin Pract. 2026 Jun 15;21:63. doi: 10.1186/s13722-026-00689-9 (PMC13501588; doi:10.1186/s13722-026-00689-9)
Supplement: Supplementary file 8 [file 13722_2026_689_MOESM8_ESM.docx]

Additional file 8. Gambling behavior at the final observation point by treatment duration and program participation.

| Treatment duration | | Group | | n (%) | | |
| --- | --- | --- | --- | --- | --- | --- |
| ≥ 3 months | | Non-participation (n=17) | | 11 (64.7%) | | |
|  |  | Participation (n=63) | | 31 (49.2%) | | |
|  |  |  |  |  |  |  |
| ≥ 6months | | Non-participation (n=13) | | 9 (69.2%) | | |
|  |  | Participation (n=54) | | 28 (51.9%) | | |
|  |  |  |  |  |  |  |
| < 3 months | | Non-participation (n=38) | | 25 (65.8%) | | |
|  |  | Participation (n=19) | | 1 (5.3%) | | |

Gambling behavior was assessed descriptively according to treatment duration and program participation at the final observation point. Data are presented as n (%).
